# Supplementary material for: Real-space imaging of acoustic plasmons in large-area graphene grown by chemical vapor deposition
Source: Nat Commun. 2021 Feb 19;12:938. doi: 10.1038/s41467-021-21193-5 (PMC7895983; doi:10.1038/s41467-021-21193-5)
Supplement: Supplementary file 1 — Supplementary Information [file 41467_2021_21193_MOESM1_ESM.pdf]

SUPPLEMENTARY INFORMATION

Real-space imaging of acoustic plasmons in large-area graphene grown by chemical vapor deposition

Sergey G. Menabde,[1,†] In-Ho Lee,[2,†] Sanghyub Lee,[3,4] Heonhak Ha,[1] Jacob T. Heiden,[1] Daehan Yoo,[2] Teun-Teun Kim,[3,5] Tony Low,[2] Young Hee Lee,[3,4,*] Sang-Hyun Oh,[2,**] and Min Seok Jang[1,***]

[1]School of Electrical Engineering, Korea Advanced Institute of Science and Technology (KAIST), Daejeon, Korea
[2]Department of Electrical and Computer Engineering, University of Minnesota, Minneapolis, USA
[3]Center for Integrated Nanostructure Physics (CINAP), Institute for Basic Science (IBS), Suwon, Korea
[4]Department of Energy Science, Sungkyunkwan University, Suwon, Korea
[5]Department of Physics, University of Ulsan, Ulsan, Korea

Email: [*]leeyoung@skku.edu; [**]sang@umn.edu; [***]jang.minseok@kaist.ac.kr
[†]These authors contributed equally to this work

S-1. AGP dispersion and its analysis

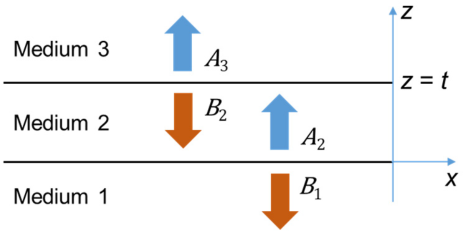

Figure S1. Schematics of a three-layer plasmonic structure. Arrows indicate the direction of exponential decay of the plasmonic mode magnetic field $H(z)$ having complex amplitudes A_m and B_m.

The model of a uniform three-layer structure with semi-infinite top and bottom layers is shown in Figure S1. The graphene layer is located at the interface between media "3" and "2" at $z = t$, where t is the spacer thickness. Assuming the TM-polarized plasmonic mode propagating in the x-direction, the *ansatz* is made that the magnetic field in each medium m = 1,2,3 takes the form $\mathbf{H}^{(m)} = \hat{\mathbf{y}} H^{(m)}(z) e^{i[k_x x - \omega t]}$, where $k_x = 2\pi/\lambda_\mathrm{p}$ is the wavevector component in the direction of propagation, and λ_p is the plasmon wavelength. The magnetic field in each layer, exponentially decaying along the z-axis, can be presented as having constant complex amplitudes A_m and B_m in the following form:

$$H^{(1)}(z) = A_1 e^{ik_z^{(1)} z} + B_1 e^{-ik_z^{(1)} z}, z < 0;$$

$$H^{(2)}(z) = A_2 e^{ik_z^{(2)}(z-t)} + B_2 e^{-ik_z^{(2)}(z-t)}, 0 < z < t;$$

$$H^{(3)}(z) = A_3 e^{ik_z^{(3)}(z-t)} + B_3 e^{-ik_z^{(3)}(z-t)}, z > t.$$

In the above equations, $k_z^{(m)} = \sqrt{k_0^2 \varepsilon_m - k_x^2}$ is the z-component of the plasmon wavevector in medium m, $k_0 = \omega/c$ is the free-space wavevector, and ε_m is the dielectric permittivity. Then, substituting the equations for the magnetic field into Maxwell's curl equation $\nabla \times \mathbf{H} - \varepsilon\varepsilon_0\, \partial\mathbf{E}/\partial t = \mathbf{J}$, we apply field continuity

conditions for electric and magnetic fields at each interface: $E_x^{(m+1)} - E_x^{(m)} = 0$ and $H^{(m+1)} - H^{(m)} = \mathrm{K}$, where $\mathrm{K}(z = 0) = 0$ and $\mathrm{K}(z = t) = \sigma(\omega)E_x$; $\sigma(\omega)$ is the optical conductivity of graphene. Taking $A_1 = B_3 = 0$ (absence of reflected or incident waves in media "1" and "3"), we obtain a system of four linear equations for the four unknowns A_m and B_m. By searching for all possible solutions of the system of equations (if any), all plasmonic eigenmodes supported by the system can be found. This powerful approach can be used to evaluate eigenmodes of multilayer structures with an arbitrary number of layers, or to calculate complex reflection and transmission coefficients in the presence of an incident wave (e.g. when $B_3 = 1$), as employed to calculate the lossy AGP dispersion in Fig. 3a in the main text.

Figure S2a demonstrates the figure of merit (FOM) for AGP and GSP modes as defined in the main text. Data for AGP in samples with 18 nm-thick (dashed) and 8 nm-thick (dash-dotted) alumina spacer is calculated for graphene carrier mobility 2000 cm^2/Vs (blue) and Fermi level –0.5 eV, corresponding to the case shown in Figs. 2b,c in the main text. For comparison, we also show FOM for both modes when carrier mobility is 500 cm^2/Vs (orange). It is evident that the AGP is always less damped than the GSP, irrespective of the carrier mobility or spacer thickness.

It must be noted that, since the same graphene parameters are employed for all calculations, the intrinsic loss in graphene is the same for both AGP and GSP modes. This leads to the similar plasmon lifetime τ = [propagation length]/[group velocity], which can be analytically or numerically obtained from the plasmon dispersion (Fig. S2b), diverging only at lower frequencies where alumina becomes lossy (due to the different fraction of the mode volume confined in alumina spacer).

Interestingly, the AGP's FOM increases as the spacer thickness decreases (Fig. S2c) despite the tighter mode confinement. This notable feature of AGP can be explained by the faster reduction of the plasmonic wavelength compared to the AGP propagation length (group velocity) as spacer thickness decreases, as has been discussed in Ref.[1] At the same time, the increasing dielectric permittivity of the medium above graphene leads to the coalescence of FOM for AGP and GSP (Fig. S2d) due to the symmetrization of the fields distribution and merging of their dispersion.

Furthermore, according to the dispersion solutions, AGP's FOM is less sensitive to the loss in graphene compared to GSP, as shown in Fig. S2e by the FOM ratio $\mathrm{FOM_{AGP}/FOM_{GSP}}$. While thinner spacer provides larger AGP's FOM due to the dispersion-related interplay between the plasmonic wavelength and propagation length, the ratio $\mathrm{FOM_{AGP}/FOM_{GSP}}$ further increases as carrier mobility in graphene decreases, implying that the more compressed AGP is less affected by the loss in graphene than the GSP. This can be explained by the AGP energy flow being mostly confined inside the dielectric spacer (Fig. S2f, bottom), in contrast to being maximal immediately near the graphene plane in case of GSP (Fig. S2f, top). This effect is even more pronounced in thinner spacers where energy flow tends to be more confined in the dielectric.

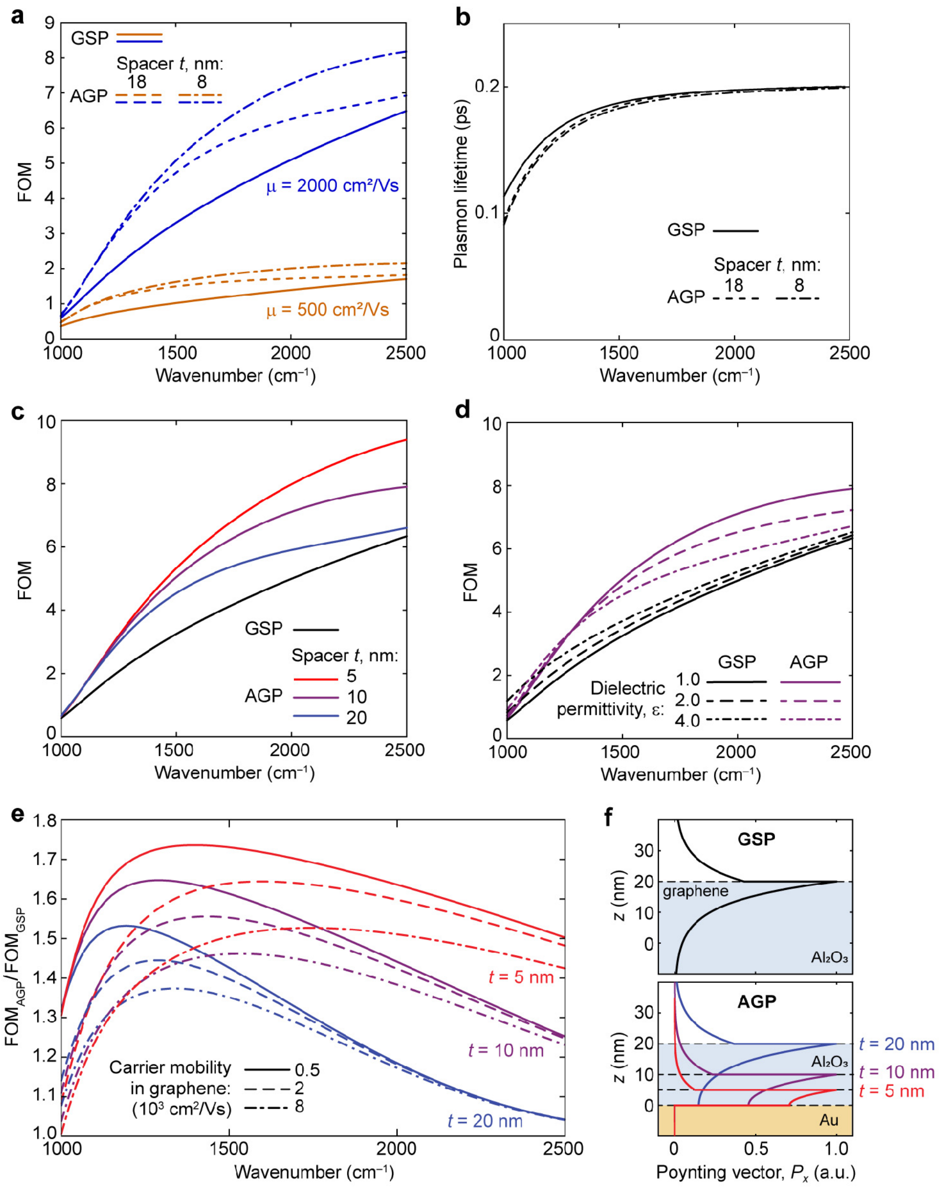

Figure S2. **a** Plasmon FOM, analytically calculated for AGP mode with spacer thickness 18 nm (dashed) and 8 nm (dash-dotted), and GSP mode on a semi-infinite alumina (solid); carrier mobility in graphene 2000 cm^2/Vs (blue) and 500 cm^2/Vs (orange). **b** Plasmon lifetime of GSP and AGP with different spacer thickness as in **a**. **c** Plasmon FOM for AGP and GSP, where AGP data is calculated for different spacer thickness. **d** FOM for GSP and AGP modes with different dielectric constant of the medium above graphene; spacer thickness is 10 nm for AGP case. **e** Ratio FOM_{AGP}/FOM_{GSP} for different spacer thickness and carrier mobility in graphene. **f** Distribution of the x-component (in the direction of propagation) of the Poynting vector for GSP (top) and AGP (bottom) with different thickness of alumina spacer t = 5, 10, and 20 nm. Results in all panels are analytically calculated assuming graphene with E_F = –0.5 eV and carrier mobility 2000 cm^2/Vs unless specified otherwise.

S-2. Impact of surface quality on AGP scattering

Figure S3 shows the atomic force microscope (AFM) scans of surfaces in the sample fabricated by the template stripping method: gold surface under the alumina spacer (Fig. S3a; after removing the alumina from the pre-fabricated sample), and the surface of alumina on top of which graphene was deposited (Fig. S3b). Naturally formed grain structure of thermally evaporated gold film have an average grain size of 80 nm. The same grain size is measured at the alumina surface, as the ALD deposition of Al_2O_3 is conformal. The RMS surface roughness a_{RMS} measured across a 1×1 μm^2 area is approximately 0.5 nm in both cases, which is very close to a typical roughness of a Si wafer 0.3 nm.

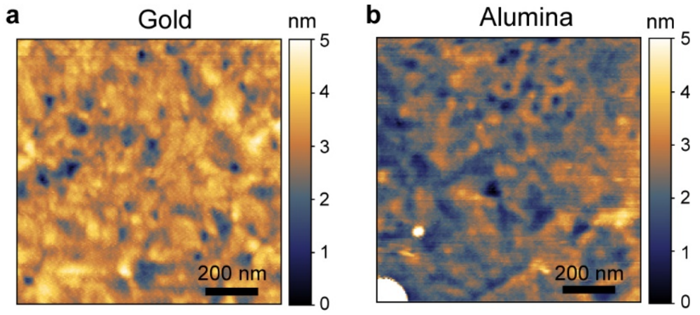

Figure S3. AFM scans of the gold and alumina surfaces between which the propagating AGP mode is confined. **a** Gold surface under the alumina layer, exposed by removing the latter after sample fabrication. **b** Topmost surface of the alumina spacer. The sample was fabricated by template stripping.

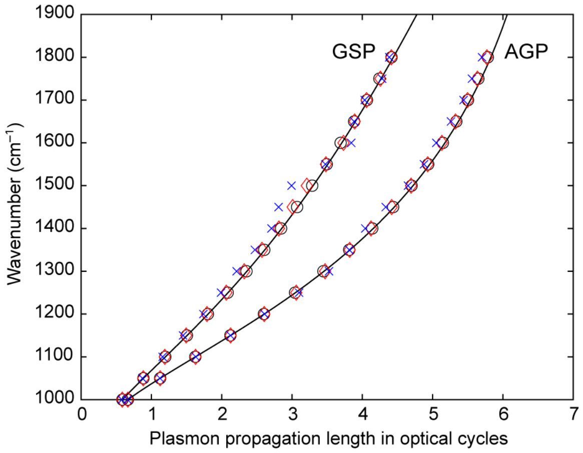

Figure S4. Normalized plasmon propagation length. Analytically (solid lines) and numerically obtained data (black circles) for flat interfaces are plotted together with numerical data for a_{RMS} = 0.5 nm (red diamonds) and 2 nm (blue crosses). Multilayer structure model parameters correspond to the case analyzed in Figs. 2b,c in the main text.

In order to estimate the AGP and GSP loss caused by scattering on rough surfaces, we performed numerical finite-element method (FEM) simulations of propagating plasmons in a uniform structure where surfaces are modelled as having a wave-like (sinusoidal) profile, with the period twice larger than the average grain size, and peak-to-peak distance given by $2\sqrt{2}a_{RMS}$. Figure S4 demonstrates the analytically calculated plasmon propagation length (solid lines) measured in optical cycles, $l = \mathrm{Re}\{q\}/(2\pi\mathrm{Im}\{q\})$, as a function of frequency for parameters corresponding to the case shown in Fig. 2b,c in the main text, and neglecting the scattering loss. Also shown are the numerically obtained l in the structure with smooth (black circles) and corrugated interfaces with a_{RMS} = 0.5 nm (red diamonds) and a_{RMS} = 2 nm (blue crosses). We find no significant change of the AGP propagation length even when the surface roughness is 2 nm, particularly at the excitation frequencies in our experiments 1000-1300 cm^{-1}. Therefore, we conclude that the scattering loss of AGP in our samples can be neglected when analyzing the near-field data.

S-3. Numerical simulation of AGP fringes at graphene edge

As explained in the main text, the AFM tip efficiently scatters only the z-component of the electric field (E_z) due to its vertically elongated geometry. Therefore, the intensity of the scattered near-field signal is directly proportional to the E_z above the sample at the position of the tip. The interaction between the point dipole source (i.e., the AFM tip of the s-SNOM) and the sample (i.e., the multilayer structure supporting AGP) can be numerically modelled by FEM or finite difference time domain (FDTD) methods in a quasi-static approximation, since the mechanical oscillation frequency of the AFM tip (~100 kHz) is several orders of magnitude lower than the optical frequencies (~10 THz). Hence, by calculating a steady-state solution for the electromagnetic fields at different tip positions, it is possible to reconstruct the near-field signal scattered by the AFM tip, $s \propto |E_z|$, where E_z is the vertical component of the electric field under the tip. In the presence on the graphene edge, probed E_z is a result of superposition between the locally induced field (due to the interaction between the tip and the sample) and the field of the propagating AGP mode (E_{AGP}) that has been reflected from the graphene edge.

Figure S5 shows the schematics of the model used to calculate the AGP fields. The actual height h_1 of the induced electric dipole over the sample is unknown, as well as the height h_2 of the tip area effectively scattering the AGP fields. Another unknown parameter is the amplitude R of the reflection coefficient at the graphene termination, which is ≈0.99 in a structure with an ideal edge. These three parameters, along with the optical conductivity of graphene, can be determined by fitting the FEM data because each combination of parameters produces a unique near-field pattern. Our calculations have provided the following model parameters corresponding to the near-field data shown in Fig. 2b in the main text: h_1 =

160 nm, $h_2 = 85$ nm, and $R = 0.8$. Since the simulations are in 2D for the sake of high speed and low resource consumption, the electric field of the reflected AGP must be adjusted to consider the energy conservation in a diverging circular wave: $E_{AGP} \propto r^{-0.5}$, where r is the propagation distance from the excitation point.

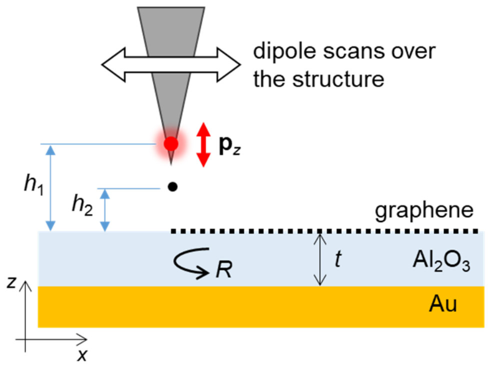

Figure S5. Numerical simulation model to calculate the near-field signal intensity detected by s-SNOM. AFM tip is simulated as a z-oriented point dipole source, and scattered field intensity is proportional to $|E_z|$ at the point under the dipole. Propagating AGP experiences reflection at the graphene edge with reflection coefficient R.

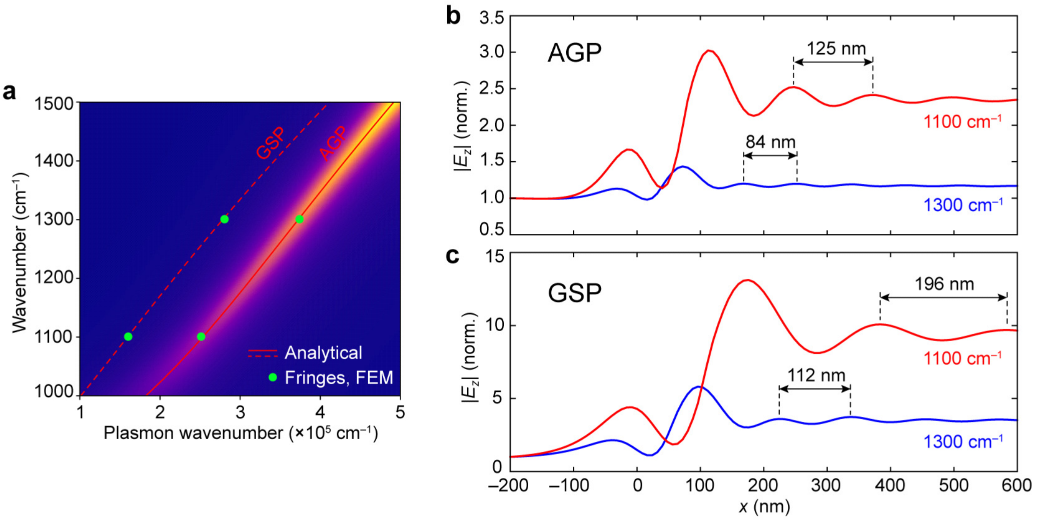

Figure S6. a Analytically calculated GSP and AGP dispersion as shown in Fig. 3a in the main text (color map and lines). Data points (green circles) correspond to the plasmonic wavelengths obtained by the numerical modelling of the interference fringes in the near-field. **b** Near-field contrast across the graphene edge, calculated as normalized $|E_z|$, for the sample case studied in Fig. 3 of the main text. **c** Near-field contrast across the graphene edge with similar properties as in **c**, for thick alumina substrate (no gold layer). Graphene edge in **b** and **c** is at $x = 0$ nm.

Similar approach can be applied to calculate the near-field profile of the GSP fringes at the graphene edge. Figure S6 demonstrates the FEM-simulated near-field contrast (i.e. $|E_z|$ normalized by that above the area without graphene) profiles across the graphene edge ($E_F = -0.46$ eV, $\mu = 2000$ cm^{-1}) on a thick alumina (for

GSP; Fig. S6c) and 21 nm alumina spacer on gold (for AGP; Fig. S6b), corresponding to the experimental case analyzed in Fig. 3 of the main text. Numerical FEM simulations for the two cases at two different frequencies provide the plasmonic wavelengths, exactly matching the analytically calculated dispersion (Fig. S6a). Note that the near-field contrast of the GSP fringes (Fig. S6c) is much higher than that of the AGP fringes (Fig. S6b). This is due to the presence of the gold layer in the AGP configuration which has high near- and far-field reflection coefficients. As we discuss in the main text, the near-field contrast reduces with the excitation wavelength as the plasmonic field is more confined. This is true for both GSP and AGP fringe patterns, as their evanescent fields above graphene behaves in similar manner at different frequencies.

S-4. s-SNOM-measured AGP dispersion at samples with gold nanoribbons

Similar to Fig. 3 in the main text, we use dispersion measurements taken over the area of interest to determine the graphene Fermi level E_F in different devices with gold nanoribbon arrays of different period. Figure S7 shows the AGP dispersion obtained from the plasmonic fringes in near-field images (circles) and the analytically calculated AGP dispersion (red lines) fitted by the corresponding value of E_F. All data is collected from the samples located at the same chip with 18 nm-thick spacer, covered with the same graphene sheet. The chip with many devices was chemically doped once, and then measured. Due to the depletion of the chemically-induced doping in time, and since the s-SNOM imaging of each device typically takes a considerable amount of time, the Fermi level of graphene is different for every set of measurements taken within days after chemical doping (as noted in Fig. S7). The obtained value of the Fermi level was then used to model the plasmonic response of the system as well as numerically calculate the near-field signal from the periodic structures.

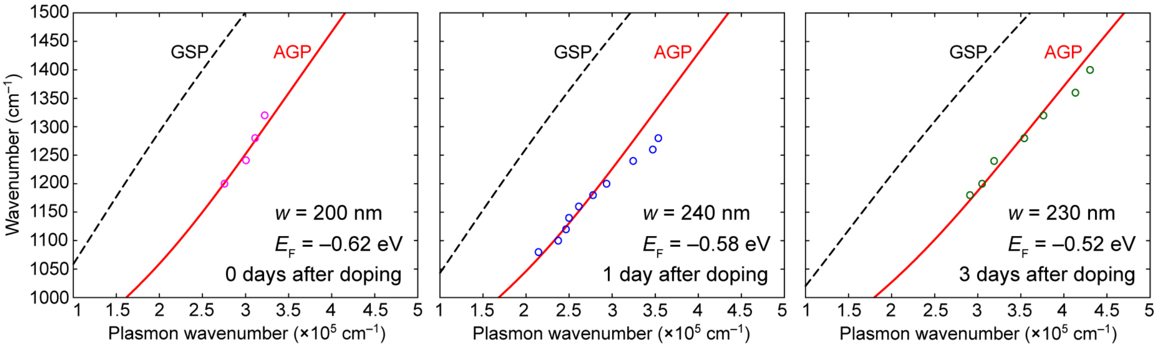

Figure S7. Analytical AGP dispersion (solid red) calculated for the fitted value of graphene Fermi level (as noted). Analytical dispersion is fitted based on the measured AGP wavelength (circles) for samples with different width of gold nanoribbons w (as noted). Also shown, the analytical GSP dispersion (dashed black) for graphene with given Fermi level when considered on a semi-infinite alumina substrate.

S-5. AGP dispersion for the propagation across the ribbons

To calculate the dispersion for AGP mode propagating across the ribbons (along the x-axis), we employ a simple model shown in Fig. S8a. In this model, the nanogap is treated as a partially reflective lateral mirror for the propagating AGP mode with complex reflection and transmission coefficients $\alpha(\omega)$ and $\delta(\omega)$, respectively. Then, the dispersion solution reduces to the eigenvalue problem for a 1D photonic crystal with the lattice constant P: $\det[\mathbf{M}_0 - e^{iKP}\mathbf{I}] = 0$, where K is the Bloch wavenumber and $\mathbf{M}_0$ is the wave-transfer matrix for the unit cell. For the model presented in Fig. S8a, the wave-transfer matrix is given by:

$$\mathbf{M}_0 = \frac{1}{\delta}\begin{bmatrix} e^{i\varphi}(\delta^2 - \alpha^2) & \alpha e^{i\varphi} \\ -\alpha e^{-i\varphi} & e^{-i\varphi} \end{bmatrix},$$

where $\varphi = wk_{\mathrm{AGP}}$ is the accumulated AGP phase over the nanoribbon width. Reflection and transmission coefficients are numerically obtained from the full-wave FEM simulations (COMSOL), where AGP is launched at a numerical port boundary and propagates over a single nanogap in the gold layer. Amplitude and phase of the reflection and transmission coefficients for lossy (realistic) case is shown in Fig. S8b by thicker lines. Note that the transmission amplitude is much larger than that of the reflection. Substituting the wave-transfer matrix into the eigenvalue problem, we obtain the following general solution for the Bloch state:

$$2\cos(KP) = \frac{e^{i\varphi}}{\delta}\left(\delta^2 - \alpha^2 + e^{-2i\varphi}\right).$$

As discussed in the main text and illustrated by Fig. 5, the band structure for the 1D periodic array of gaps does not show any bandgap (Fig. S8c, left), corresponding to the general case of polaritonic dispersion solved for the complex wavevector and real frequency, which does not exhibit a "true" bandgap unless the system is lossless[2].

We also calculated the structure with tuned-down losses in all materials: with the carrier mobility in graphene 20,000 cm^2/Vs, purely real alumina permittivity, and gold permittivity calculated according to the Drude-Sommerfeld model[3] with electron relaxation time 100 times larger than the reported experimental value of 14 fs. These assumptions practically do not affect the reflection and transmission coefficients at the nanogap (Fig. S8b; thinner lines). The resulting band structure for the case of negligible material loss is shown in Fig. S8c (middle), which also does not show bandgap opening due to the following reason. There exist two distinctive loss channels in our structure: non-radiative losses due to the ohmic loss in gold and graphene, and scattering losses at the nanogaps between the gold stripes. Therefore, due to the existence of the scattering losses, the resulting dispersion relation with negligible propagation loss still shows connected bands, although the distortion of the bands becomes pronounced (both in the real and imaginary wavenumbers) compared to the case with full losses.

Since the scattering loss at the nanogap depends on the geometry of the structure, it is impossible to simply turn it off in rigorous full-wave simulations. Nevertheless, it is still possible to artificially model the truly lossless system by taking the transmission coefficient from the rigorous simulations and setting the reflection coefficient in a way that the system satisfies the energy conservation and reciprocity (i.e. when $|\alpha|^2 + |\delta|^2 = 1$ and $\delta/\delta^* = -\alpha/\alpha^*$). In this case, using the same waveguide parameters as in the low-loss case, the Bloch solution reveals the presence of bandgaps with vanishing $\mathrm{Re}\{K_x\}$ (Fig. S8c, right).

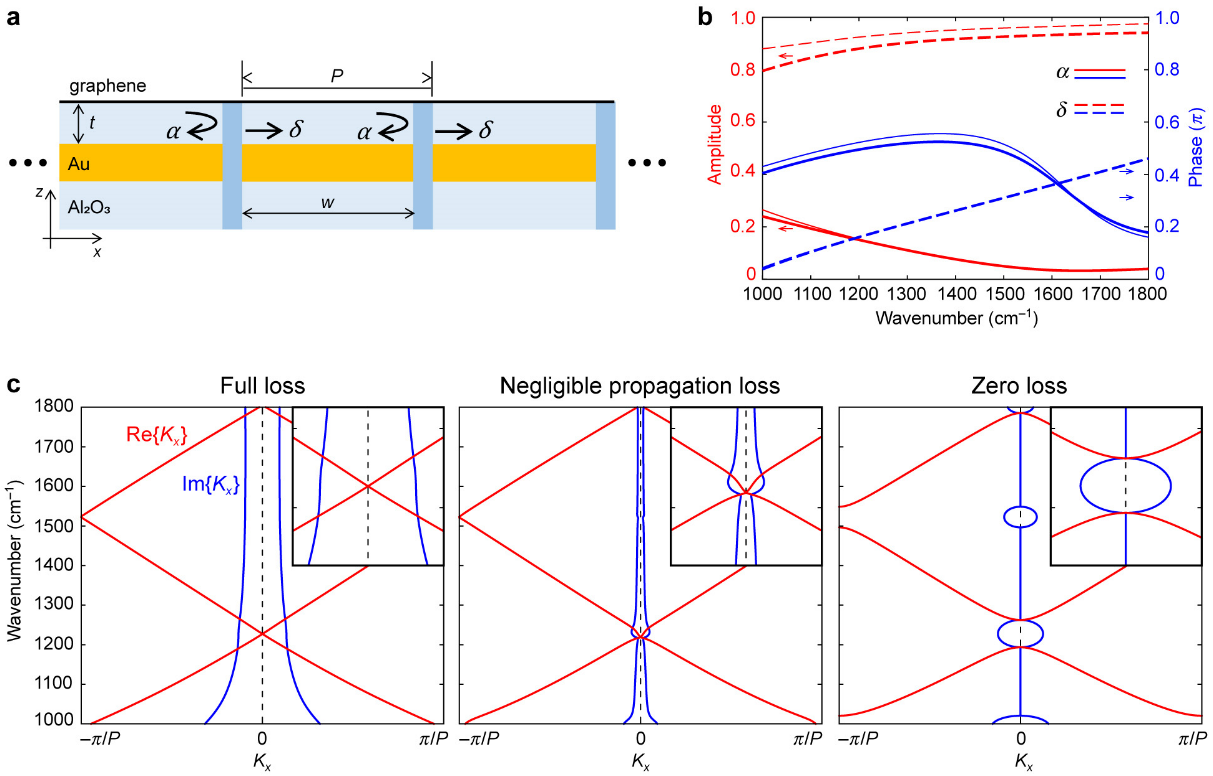

Figure S8. a Model of a 1D photonic crystal configuration for AGP where the nanogap between gold bars is treated as an element with complex reflection and transmission coefficients $\alpha(\omega)$ and $\delta(\omega)$, respectively. **b** Numerically calculated $\alpha(\omega)$ (solid) and $\delta(\omega)$ (dashed) of the 30 nm-wide nanogap in a 20 nm-thick gold layer: amplitude (red) and phase (blue). Thick lines correspond to real lossy materials, while thin lines correspond to the materials with negligible loss. **c** AGP band diagram plotted for the real (red) and complex (blue) parts of the Block wavenumber K_x in the 1D array of nanogaps: actual lossy materials (left; shown in the main text), materials with negligible loss (middle), and the mathematical model for lossless structure (right); the insets show the close-up of the center of the Brillouin zone where the far-field coupling is accessible.

S-6. Near-field profile over nanoribbons array at higher frequencies

Figure S9 demonstrates the distribution of near-field amplitude s and phase φ over the sample with $P = 260$ nm ($w = 230$ nm) shown in Fig. 5c in the main text, but at higher excitation frequencies of 1240 cm^{-1} (Fig. S9a) and 1400 cm^{-1} (Fig. S9c). At these frequencies, the scattering of the AGP at the gold ribbon edges intensifies, and may contribute to new features in the near-field profiles across the ribbons (solid black in Fig. S9b,d). These features are not observed at the simulated data plots (dashed red in Fig. S9b,d), obtained by the 2D full-wale simulations with a source being a line of dipoles, without adjusting the AGP electric field amplitude for a circular diverging wavefront as has been done for the analysis of interference fringes at the graphene edge in section S-3. Note that the position of the field maxima/minima is different in each case. No far-field source is used in simulations.

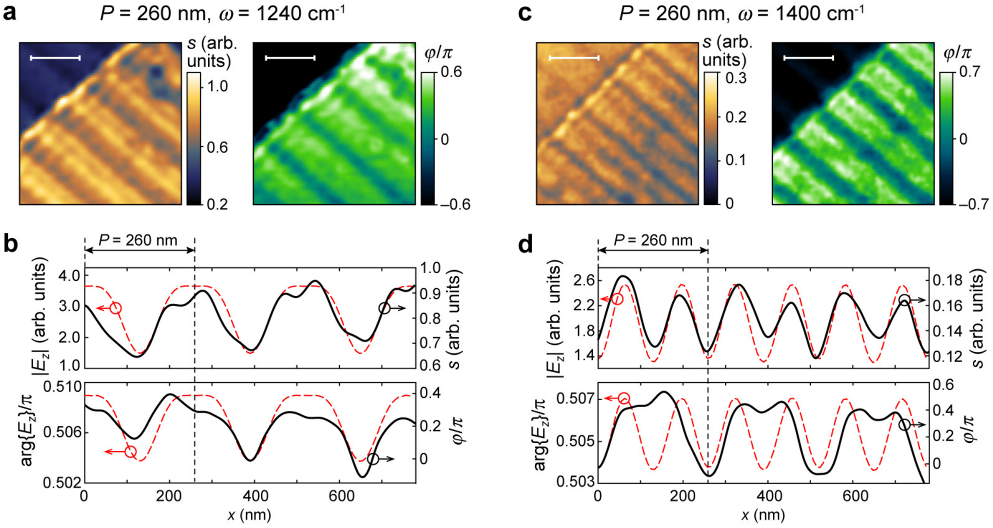

Figure S9. a Near-field signal amplitude $s(x,y)$ and phase $\varphi(x,y)$ measured over the sample with $P = 260$ nm ($t = 18$ nm, $E_F = -0.52$ eV, $\lambda_{AGP} \approx 225$ nm) at $\omega = 1240$ cm^{-1} when the AGP momentum k_{AGP} is larger than the array momentum $2\pi/P$. **b** Profiles of $s(x,y)$ (top panel) and $\varphi(x,y)$ (bottom panel) across the nanoribbons (black solid) corresponding to the scans shown in **a**, and the numerically obtained $|E_z|$ (top panel) and $\arg\{E_z\}$ (bottom panel) from the FEM simulation of the near-field measurement; parameters of the tip model are the same as in Fig. 2c of the main text. **c**,**d** Same as in **a**,**b** but measured at $\omega = 1400$ cm^{-1} when the AGP momentum is significantly larger than the array momentum. Scale bars are 300 nm.

In order to better understand the mixed near-field signal at high frequencies, it is instructive to analyze the eigenmodes in the periodic array of nanoribbons. The infinite 1D array of nanoribbons is modelled as a unit cell of size P with periodic boundary conditions in x-direction (Fig. S10a). Eigenmode analysis at the frequencies of interest reveals three AGP modes propagating along the ribbons, illustrated by their field distribution in Fig. S10a-c. The "fundamental" mode (Fig. S10a) possesses the wavenumber $\beta_y^{(a)}$ almost

equal to that of the AGP in a uniform structure β_{AGP}, as demonstrated by the dispersion plot in Fig. S10d. Upon the reflection at the graphene edge, this mode forms the interference fringes, revealing the AGP wavelength at given frequency (Fig. 4b,c in the main text). The second-order array mode has two branches with different field distribution across the unit cell (Fig. S10b,c). The mode with slightly larger wavenumber $\beta_y^{(b)}$ does not couple to the nanogaps (Fig. S10b), while the other mode with $\beta_y^{(c)}$ is coupled to the metal gaps (Fig. S10c) and therefore, is more lossy. Due to their higher loss, the high-order modes are difficult to observe upon the far-field excitation, while expected to be efficiently excited in near-field. The Bloch mode's dispersion $K_x(\omega)$ (see Section S-5 for details) is shown as a red line in Fig. S10d, and practically does not differ from β_{AGP} and $\beta_y^{(a)}$. The efficient far-field coupling to the Bloch mode is expected only at the phase-matching in the array as shown on the inset in Fig. S10d, which corresponds to the near-field images shown in Fig. 5a,b in the main text.

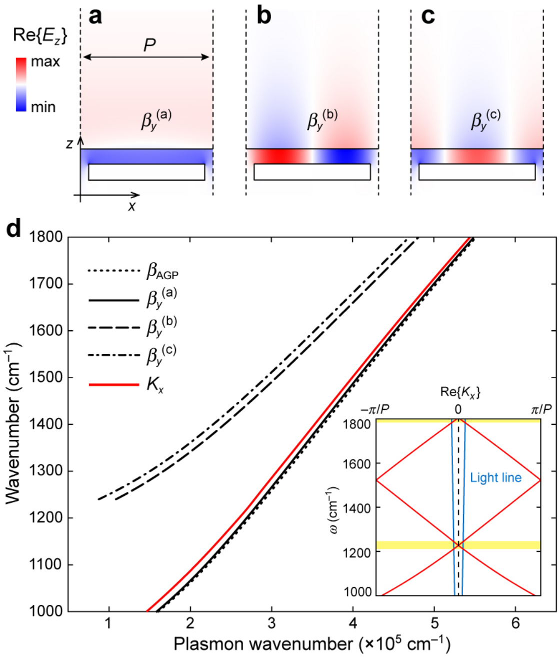

Figure S10. a-c E_z field distribution of AGP eigenmodes propagating along the nanoribbons in y-direction, obtained with the FEM eigenmode solver. **d** Numerically obtained dispersion of the AGP propagating along (black) and across (red) the nanoribbons axes; array parameters $P = 230$ nm and $E_F = -0.62$ eV correspond to the topical case analyzed in Fig. 5a,b in the main text. β_{AGP} is the AGP wavenumber in the uniform structure, and K_x is the Bloch wavenumber. Inset: band diagram corresponding to the full loss case in Fig. S8c, indicating the narrow frequency band where coupling to free-space light is possible (yellow stripe); note that this phase-matching frequency marks the cutoff for the second-order AGP mode.

Considering the calculated eigenmodes in the array, we attribute the periodic near-field signal across the ribbons at phase-matching (Fig. 5a in the main text) to the excitation of the Bloch state, resembling that in

a periodic array of partially reflective mirrors where the field amplitude is maximal in the middle of the unit cell. When frequency is detuned from the phase-matching in the array, Bloch mode is no longer dominant, and the near-field signal contains contribution from other modes in the array. However, detailed analysis of the near-field images in the presence of multiple modes and scattering sources is a non-trivial task and is left for future investigation.

REFERENCES

1 Voronin, K. V. *et al.* Nanofocusing of acoustic graphene plasmon polaritons for enhancing mid-infrared molecular fingerprints. *Nanophotonics* **9**, 2089-2095 (2020).

2 Huang, K. C. *et al.* Nature of lossy Bloch states in polaritonic photonic crystals. *Phys. Rev. B* **69**, 195111 (2004).

3 Kischkat, J. *et al.* Mid-infrared optical properties of thin films of aluminum oxide, titanium dioxide, silicon dioxide, aluminum nitride, and silicon nitride. *Appl. Optics* **51**, 6789-6798 (2012).
